# Multiplicity of *bla*_KPC_ Genes and pKpQIL Plasmid Plasticity in the Development of Ceftazidime-Avibactam and Meropenem Coresistance in Klebsiella pneumoniae Sequence Type 307

**DOI:** 10.1128/aac.00368-23

**Published:** 2023-07-10

**Authors:** Gabriele Arcari, Riccardo Polani, Stefania Santilli, Valerio Capitani, Federica Sacco, Francesco Bruno, Aurora Garcia-Fernandez, Giammarco Raponi, Laura Villa, Giuseppe Gentile, Alessandra Carattoli

**Affiliations:** a Department of Molecular Medicine, Sapienza University of Rome, Rome, Italy; b Complex Operating Unit of Microbiology and Virology, Policlinico Umberto I, Rome, Italy; c Department of Infectious Diseases, Istituto Superiore di Sanità, Rome, Italy; d Department of Public Health, Sapienza University of Rome, Rome, Italy; e Department of Translational and Precision Medicine, Sapienza University of Rome, Rome, Italy

**Keywords:** plasmid sequencing, KPC-31, carbapenems, ceftazidime-avibactam, antimicrobial resistance, ST307, high-risk clone, *Klebsiella pneumoniae*, plasmid

## Abstract

In 2021, Klebsiella pneumoniae sequence type 307 (ST307) strains causing pulmonary and bloodstream infections identified in a hospital in Rome, Italy, reached high levels of resistance to ceftazidime-avibactam (CZA). One of these strains reached high levels of resistance to both CZA and carbapenems and carried two copies of *bla*_KPC-3_ and one copy of *bla*_KPC-31_ located on plasmid pKpQIL. The genomes and plasmids of CZA-resistant ST307 strains were analyzed to identify the molecular mechanisms leading to the evolution of resistance and compared with ST307 genomes at local and global levels. A complex pattern of multiple plasmids in rearranged configurations, coresident within the CZA-carbapenem–resistant K. pneumoniae strain, was observed. Characterization of these plasmids revealed recombination and segregation events explaining why K. pneumoniae isolates from the same patient had different antibiotic resistance profiles. This study illustrates the intense genetic plasticity occurring in ST307, one of the most worldwide-diffused K. pneumoniae high-risk clones.

## INTRODUCTION

Klebsiella pneumoniae is one of the most frequent causes of nosocomial infections, including sepsis, liver abscess, and pneumonia, and represents a major problem for health systems at the global level (1). High-risk clones of this species are characterized by rapid evolution and adaptation to antimicrobial drugs, leading to extensively drug-resistant phenotypes (2, 3).

In the early 2000s, carbapenem resistance emerged in K. pneumoniae at a global level by the spread of KPC carbapenemase-producing strains of clonal group 258 (CG258), including sequence types 258 (ST258) and ST512 (4–6).

The ST307 clone was traced back as a globally diffused high-risk clone from the early to mid-2000s (7, 8). Genomic studies on early K. pneumoniae ST307 isolates showed that this clone was characterized by the presence of IncF plasmids called pKPNs encoding the CTX-M-15 extended spectrum beta-lactamase (ESBL), conferring cephalosporin resistance (8), but the first KPC-producing strains emerged only in 2008 (9). They were initially described in Italy, Colombia, and the United States (9, 10). In each country of isolation, the most frequent KPC variant of its respective plasmid type (i.e., IncF pKpQIL-like plasmids [6] encoding KPC-3 in Italy and IncN plasmids encoding KPC-2 in Colombia) moved from other K. pneumoniae STs into ST307 (10). This event occurred independently in different countries after the spread of CTX-M-15-producing ST307, as was inferred by the fact that strains had distinct KPC plasmids but related pKPN-like plasmids carrying *bla*_CTX-M-15_ (10). On pKPN, up to five putative virulence clusters were identified: the *lac* operon, the Fec-like iron (III) dicitrate and the glutathione ABC-transport systems, the urea transport system, and the cluster for glycogen synthesis. ST307 also carried the yersiniabactin siderophore, mobilized by an integrating conjugative element (ICE) (8, 10, 11).

In 2016, a novel combination of a third-generation cephalosporin (ceftazidime), with the non-beta-lactam beta-lactamase inhibitor (avibactam), was introduced into clinical practice to treat infections by carbapenem-resistant *Enterobacterales* isolates producing class A and D carbapenemases (12).

In 2019, we described the emergence of the first ST307 strains showing ceftazidime-avibactam (CZA) resistance at the University Hospital Policlinico Umberto I (PUI) in Rome, Italy (13). The *bla*_KPC-31_gene located on the Tn*4401* transposon, was the most frequent (11 strains of 32) CZA resistance mechanism identified in that study. Among KPC-31 producers, ST307 (5/11) and ST101 (4/11) were the most represented STs, while in 17 CZA-resistant ST512 strains, 6 different KPC variants were observed, and only one strain produced KPC-31. KPC-31 is characterized by a single amino-acid substitution (a D-to-Y change at position 179 [D179Y]) that confers CZA resistance at the expense of carbapenemase activity (14). Therefore, the CZA-resistant ST307 strains identified in 2019 were susceptible to carbapenems.

In 2021, despite epidemiological association linking the cases, a KPC-31-producing ST307 isolate identified at the PUI showed interesting differences in resistance levels to CZA and carbapenems. In this study, we describe the CZA resistance mechanisms and plasmid evolution that occurred in contemporary ST307 strains, compared with the genomes of historical strains isolated from the same hospital and with a selection of ST307 genomes from the public NCBI data set.

## RESULTS

### Genomics of ST307 strains.

Three CZA-resistant strains (0213-2021, 1001-2021, and 0323-2021) isolated from two patients hospitalized at PUI were subjected to Illumina and Oxford Nanopore Technologies (ONT) sequencing and assigned by *in silico* multilocus sequence typing (MLST) to ST307 (Table 1). These genomes were compared with 6 genomes of ST307 strains (1802, 1203, 0603, 21, 27B, and 3) isolated in 2019 at the same hospital and with 30 ST307 genomes downloaded from NCBI GenBank (see Data set S1 in the supplemental material).

**TABLE 1:** MICs of Klebsiella pneumoniae ST307 analyzed in this study

Strain	Date (mo/day/yr)	KPC	Ward^*a*^	Source^*b*^	Mutation in:^*c*^		MIC (mg/L) for:^*d*^		
					OmpK35	OmpK36	MEM	CZA^*d*^	CAZ^*d*^
0213-2021	02/13/21	KPC-31	NEU	RTS	WT	WT	<0.12	**12**	**>32**
1001-2021	04/03/21	KPC-31	NEU	RTS	WT	ΔOmpK36 [pG180fs]	8	**24**	**>32**
0323-2021	03/23/21	KPC-3-KPC-31	NEU	BC	WT	WT	**>16**	**32**	**>32**
0603	03/06/19	Neg	HEM	RS	WT	ΔOmpK36 [pD47fs]	4	0.75	**>32**
1802	02/18/19	Neg	HEM	RS	ΔOmpK35 [pG75fs]	ΔOmpK36 [pG149fs]	8	4	**>32**
1203	03/12/19	Neg	HEM	RS	ΔOmpK35 [pG75fs]	ΔOmpK36 [pM57startcodon]	**16**	2	**>32**
27B	03/12/19	KPC-31	HEM	AF	WT	WT	<0.12	**12**	**>32**
21	10/10/19	KPC-31	MED	RS	WT	WT	<0.12	**12**	**>32**
3	02/18/19	KPC-3	ICU	BC	ΔOmpK35 [pG75fs]	WT	**16**	0.75	**>32**

a ICU, intensive care unit; MED, medical clinics; HEM, hematology wards; NEU, neurosurgical unit.

b BC, blood culture; RS, rectal swab; RTS, respiratory tract sample; AF, ascitic fluid.

c ΔOmpK35 and ΔOmpK36 indicate predicted premature termination of translation due to nonsense and frameshift (fs) mutations occurring in the OmpK35 and OmpK36 proteins. WT, wild-type porin sequences.

d CZA MICs were determined using the CZA gradient test (Liofilchem). MEM, meropenem; CAZ, ceftazidime; CZA, ceftazidime-avibactam. Numbers in bold indicate MICs interpreted as resistant.

**FIG 1:**
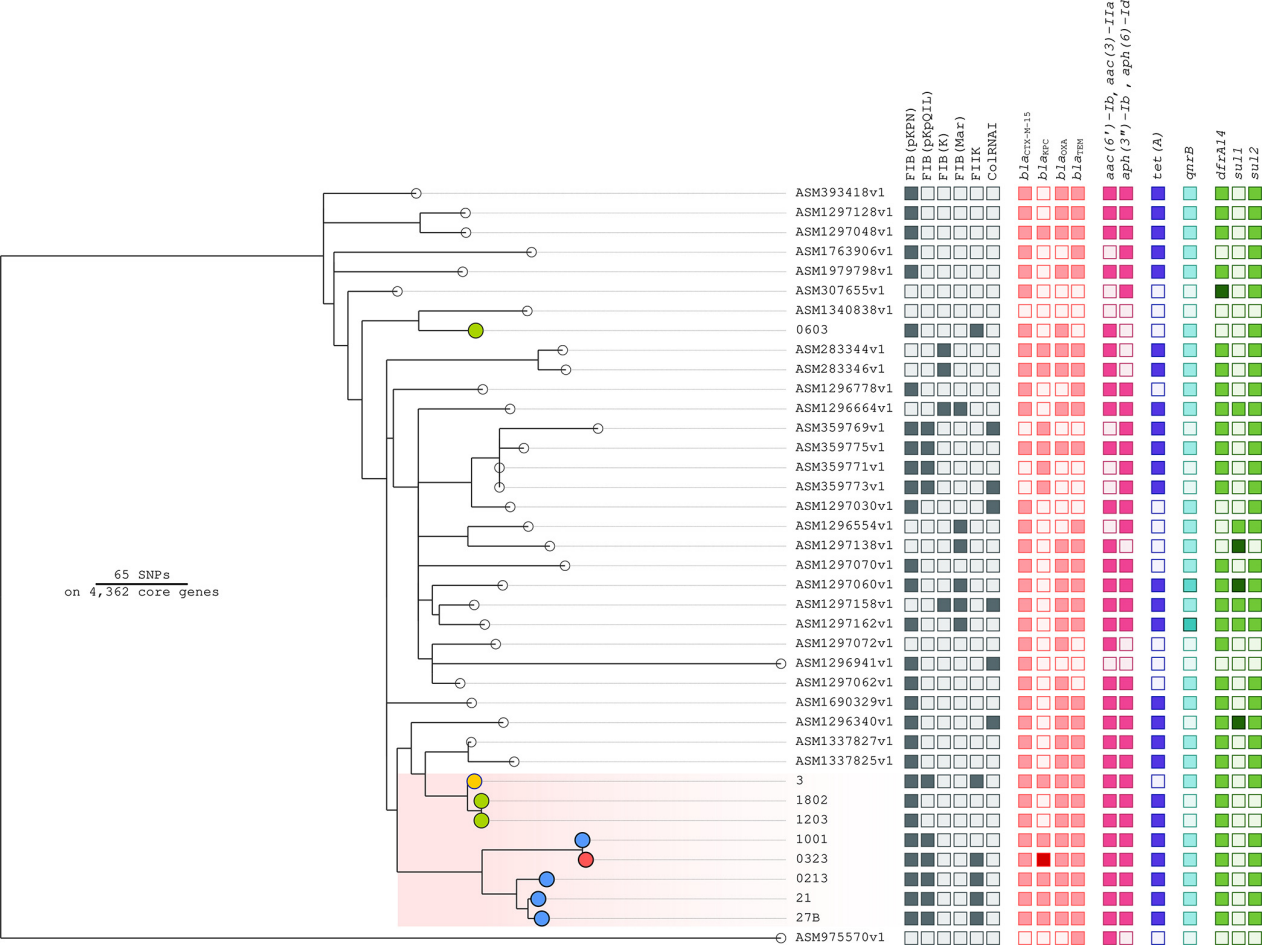
Phylogenetic tree based on a core genome alignment of Klebsiella pneumoniae ST307 isolates. The figure shows an unrooted maximum likelihood phylogenetic tree based on a concatenated core gene alignment (4,362 genes) of 39 K. pneumoniae genomes belonging to ST307. Of the 39 genomes, 30 (indicated by empty circles) were downloaded from GenBank, 3 genomes (3, 21, and 27B) were already described (13), and 6 genomes (0213-2021, 0323-2021, 1001-2021, 1202, 1803, and 0603) were obtained in this study. Carbapenem-susceptible strains 1802, 1203, and 0603 are indicated by green dots, the KPC-3-producing strain 3 is indicated by a yellow dot, CZA-resistant KPC-31-producing strains are identified by blue dots, and the CZA-MEM–resistant strain 0323-2021 is indicated by a red dot. The colored squares indicate the presence and empty squares the absence of the feature in the respective metadata column. Darker colors indicate the presence of multiple copies of the feature in the respective genome. Colored squares in the data set report the major resistance genes and replicons: gray, replicons; pink, beta-lactamase; magenta, aminoglycoside resistance; blue, tetracycline resistance; cyan, quinolone resistance; and green, sulfonamide resistance.

A midpoint-rooted phylogenetic tree, based on 4,362 core genes, showed at least three different ST307 lineages (separated by >60 single nucleotide polymorphisms [SNPs]) circulating in the hospital in the 2019–2021 period (Fig. 1). One lineage included the carbapenem-susceptible strains 1802 and 1203 and the KPC-3-producing strain 3, differing from each other by only 6 SNPs. A second lineage included the CZA-resistant strains identified at the hospital. Strain 0213-2021 was related to 21 and 27B (15 SNPs). Strains 1001-2021 and 0323-2021, isolated from the same patient, were highly related to each other (1 SNP difference in the core genome) but differed by 45 to 77 SNPs from the other CZA strains (Fig. 1). Both lineages carried the yersiniabactin (*ybt*) siderophore sequence type 384 (YbST384) (11), mobilized by the integrating conjugative element ICE*Kp*4. On the same branch with these two lineages, there were 2 genomes of OXA-48-producing isolates (ASM1337825 and ASM1337827) from pets in Switzerland (15) and one from South Korea (ASM1296340). These isolates also carried the YbST384 locus.

The genome of the susceptible strain 0603 represented the third lineage at the hospital, with a mean distance of 81 SNPs. It carried a different YbST157 *ybt* siderophore, mobilized by an ICE*Kp*3 integrating conjugative element. The isolate showing the closest phylogenetical relationship with 0603 was sampled in Rome at a different hospital and did not carry any virulence determinant (ASM1340838).

### Resistome and mobilome of ST307 strains.

No carbapenemase genes were present in strains 1802, 1203, or 0603, showing reduced susceptibility to both CZA and MEM. These isolates carried several antimicrobial resistance genes (Data set S2), including *bla*_CTX-M-15_. In 1802 and 1203, this gene is located on indistinguishable pKPN plasmids (GenBank accession number OM489434), but pKPN in 1203 is integrated into the chromosome (OM489428) (Table 2; Fig. S1).

**TABLE 2:** Genetic characteristics of pKpQIL and pKPN plasmids identified in Klebsiella pneumoniae ST307

Strain	Data for gene:			
	*bla*_KPC_ ^*a*^		*bla*_CTX-M-15_	
	Location (plasmid no. of copies per cell)	Size (kb)	Location (no. of copies)	Size (kb)
1802	Neg		pKPN (0.95)	156
1203	Neg		pKPN in the chromosome	
21	*bla*_KPC-31_ on pKpQIL-pKPN (1.4)	255	pKpQIL-pKPN (1.4)	255
27B	*bla*_KPC-31_ on pKpQIL-pKPN (1.71)	238	pKpQIL-pKPN (1.71)	238
0213-2021	*bla*_KPC-31_ on pKpQIL (1.97)	114	pKPN (1.50)	153
0323-2021	*bla*_KPC-3_ (2 copies), *bla*_KPC-31_ on pKpQIL (2.13)	136	pKPN (1.25)	140
1001-2021	*bla*_KPC-31_ on pKpQIL (1.73)	83	pKPN (1.23)	140
3	*bla*_KPC-3_ on pKpQIL (0.85)	113	pKPN (1.52)	151
0603	Neg		Chromosome	

a Neg, no *bla*_KPC_ present. Numbers of complete, circular copies of plasmids pKpQIL and pKPN analyzed in this study were measured using the Unicycler tool (19).

CZA-resistant ST307 strains carry *bla*_KPC-31_, located on transposon Tn*4401a* in pKpQIL-like plasmids. Strains 21 and 27B carried the gene on a large plasmid composed of a fusion of pKPN and pKpQIL, as previously described (13). Comparison of the original pKpQIL (GenBank accession number GU595196) (6) and pKpQIL in strain 3 (99.82% nucleotide identity; 100% coverage), showed several nucleotide mutations: 63 SNPs in the *traC* gene (positions 77821 to 80440; ON002623.2), causing 9 amino acid substitutions in the deduced TraC protein sequence; 14 SNPs in the *traD* gene (positions 61389 to 61586; ON002623.2), causing 3 amino acid substitutions in the deduced TraD protein sequence; and other SNPs identified in 5 genes encoding hypothetical proteins. The *traC* SNPs were observed only in pKpQIL of strain 3, while the *traD* gene allele was conserved in all ST307 pKpQIL sequences analyzed in this study.

**FIG 2:**
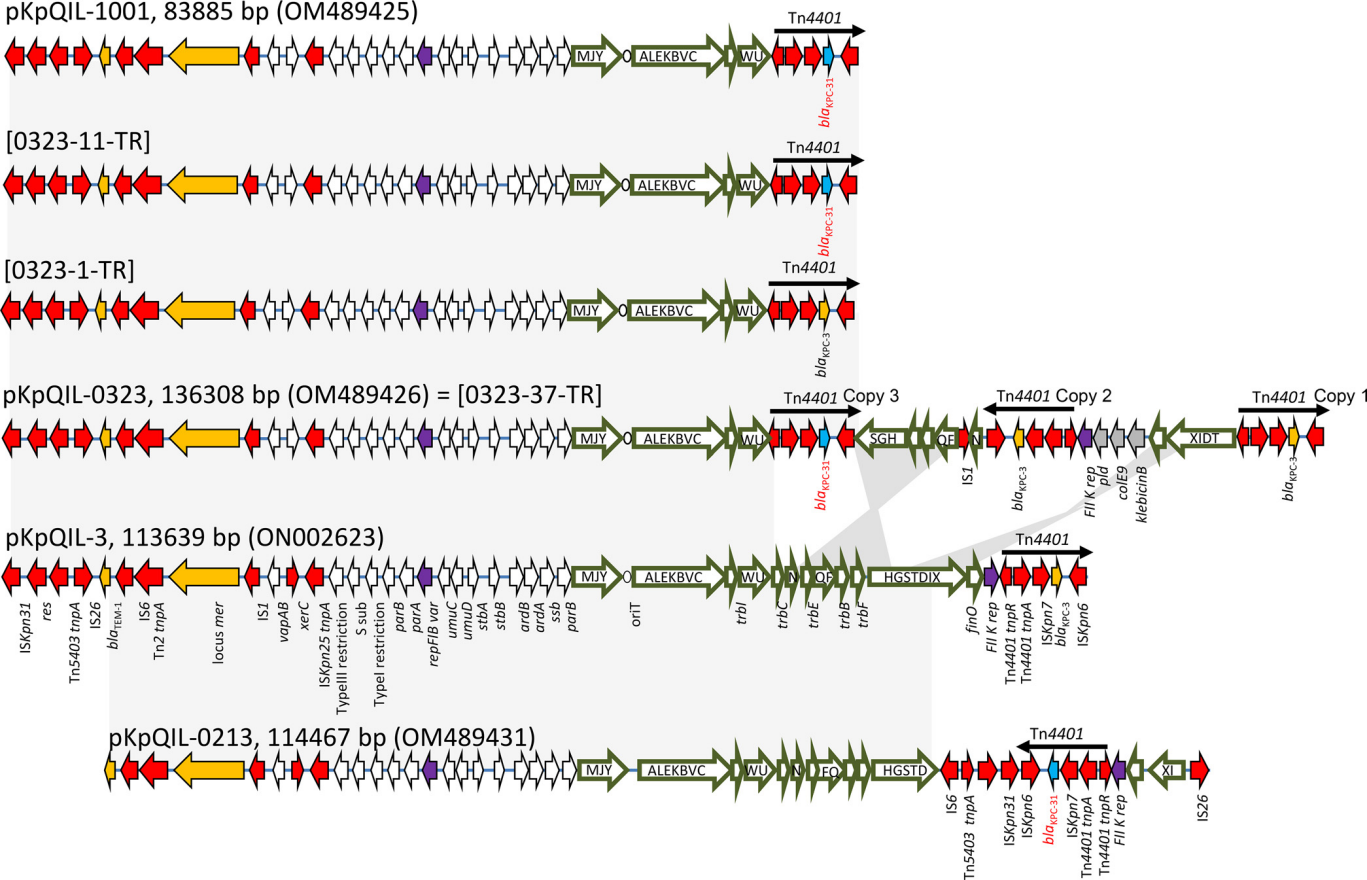
Maps of pKpQIL-307 plasmids. The major structural features of pKpQIL-1001, pKpQIL-0323, pKpQIL-3, and pKpQIL-0213 identified in ST307 K. pneumoniae isolates 1001-2021, 0323-2021, 3, and 0213-2021, respectively, and maps of the three types of plasmids obtained by transformation from strain 0323-2021, indicated as [0323-37-TR], [0323-1-TR], and [0323-11-TR]. Closely related regions are shadowed (>97% nucleotide identity). Predicted coding sequences are indicated by colored arrows oriented in the direction of transcription of each respective gene: resistance genes, yellow; *bla*_KPC-31_, blue; transposon-related genes and insertion sequences, red; replicons, violet; klebicin cluster, gray; and other genes, white. The arrows outlined in dark olive indicate the *tra* locus (each conjugation gene is indicated by a capital letter within the olive arrow) and the associated *trb* and *finO* genes. *oriT* is represented by a white circle. The sizes of the arrows are not to scale.

Recent CZA-resistant ST307 isolates showed rearranged versions of pKpQIL. In particular, in 0213-2021, an IS*26*-mediated inversion of the region, including the Tn*4401a*::*bla*_KPC-31_, FII(K), *finO*, *traX*, and *traI* genes, was observed, and the pKpQIL transfer locus was truncated at the *traD* gene (Fig. 2). Isolate 1001-2021 also carried a truncated version of pKpQIL (pKpQIL-1001), deleted at the *traU* gene, carrying the Tn*4401a*::*bla*_KPC-31_. Comparison of the original pKpQIL (GenBank accession number GU595196) (6), pKpQIL-0213, and pKpQIL-1001 highlighted striking similarities between the plasmids (100% nucleotide identity), with a deletion of 29,752 bp in pKpQIL-1001.

Strain 0323-2021 produced ambiguous hybrid Illumina and ONT read assemblies, including a version of pKpQIL carrying three copies of the *bla*_KPC_ gene, two copies of *bla*_KPC-3_, and one copy of *bla*_KPC-31_. The complete plasmid assembly required further evaluation, as described below.

In 0603, wild-type OmpK35 and OmpK36 were detected. In 1203 and 1802, frameshift mutations were present in the deduced protein sequences of OmpK35 and OmpK36, predicting premature termination of translation. All CZA-resistant ST307 strains showed wild-type OmpK35 and OmpK36 porins, except strain 1001-2021, where a frameshift mutation in the deduced OmpK36 protein sequence predicted the premature termination of translation (Table 2).

### Analysis of plasmids in the ST307 0323-2021 CZA-MEM–resistant strain.

Because of the complexity of the repeated elements in strain 0323-2021, the hybrid Illumina and ONT read assembly needed experimental confirmation. ONT was applied to the original K. pneumoniae strain 0323-2021 using purified plasmid DNA as the template. Running ONT on a good plasmid extract, the read length graph showed highly represented reads covering the entire plasmid length (Fig. S2). At least three reads visible as higher bars in the graph were interpreted as plasmids of 83 kb, 136 kb, and 140 kb, coexisting in this K. pneumoniae strain.

To discern these plasmids, purified plasmid DNA from the K. pneumoniae strain was used to transform chemically competent Escherichia coli DH5-α cells. The following three types of transformants were obtained from strain 0323-2021 (Fig. 2; Table S2).

1. Type 1 transformants (prototype 0323-37-TR) showed MEM and CZA resistance. ONT sequencing showed a complete, circular plasmid of 136 kb, carrying 2 copies of *bla*_KPC-3_ and 1 copy of *bla*_KPC-31_ (pKpQIL-0323). One of the two *bla*_KPC-3_ copies was in the Tn*4401a* identified in the same position as in pKpQIL-3 (copy 1 in Fig. 2). The second Tn*4401a*::*bla*_KPC-31_ (copy 2) was inserted in the opposite orientation as copy 1, downstream of the *traU* gene. The third Tn*4401a*::*bla*_KPC-3_ copy (copy 3) was integrated in the same orientation as copy 1 in the *traS* gene. We hypothesize that recombination between copies 1 and 2 in opposite orientations caused the inversion of the pKpQIL region comprising the *traT*, *traD*, *traI*, *traX*, and *finO* genes up to the FII(K) replicon. Moreover, recombination between copy 2 and copy 3 could have led to the inversion of the transfer locus genes downstream of the *traU* integration site (genes *traS* to *traN*). The 5-bp target-site duplications (TSDs) that originated by transposition of the Tn*4401a* copies were identified (Fig. S3). To complete the plasmid analysis, the location of the Tn*4401a*::*bla*_KPC-3_ and Tn*4401a*::*bla*_KPC-31_ copies in pKpQIL-0323 was also confirmed by PCR and Sanger sequencing of the amplicons, using primers designed in the *traS*, *traN*, and *bla*_KPC_ genes (Table S1). In pKpQIL-0323, the klebicin B-encoding genes were inserted between the *finO* gene and the FII(K) replicon. These genes were not present in other genomes of our collection (Fig. 2).
2. Type 2 transformants (prototype 0323-1-TR) showed MEM resistance and CZA susceptibility. In these transformants, a plasmid of 83 kb was detected, carrying a single copy of the Tn*4401a*::*bla*_KPC-3_. This plasmid was probably generated by the recombination of pKpQIL-0323, which occurred between the directly oriented Tn*4401a*::*bla*_KPC-3_ and Tn*4401a*::*bla*_KPC-31_ copies 1 and 3, resulting in the loss of the Tn*4401a*::*bla*_KPC-31_ copy and the deletion of the pKpQIL backbone at the *traU* gene.
3. Type 3 transformants (prototype 0323-11-TR) showed MEM susceptibility and CZA resistance. In these transformants, the same 83-kb plasmid identified in the type 2 transformants was observed, but the recombination between the two Tn*4401a*::*bla*_KPC-3_ and Tn*4401a*::*bla*_KPC-31_ copies resulted in the maintenance of a single Tn*4401a*::*bla*_KPC-31_ copy and the deletion of the backbone at the *traU* gene. This plasmid was identical to the 83-kb plasmid identified in K. pneumoniae strain 1001-2021 (Fig. 2).

These results demonstrate that the 83-kb and 136-kb versions of pKpQIL were coresident within K. pneumoniae strain 0323-2021. The higher 140-kb read observed in the ONT profile (Fig. S2) was interpreted as pKPN, also coresident with the two pKpQIL variants in this K. pneumoniae strain.

## DISCUSSION

This study highlights the consequences of the strong CZA and MEM selective pressure on K. pneumoniae ST307. This high-risk clone shows sharp plasticity of plasmids circulating in multiple lineages, flowing within the hospital.

Reduced susceptibility to MEM in ST307 ancestor strains 1802 and 1203, negative for carbapenemase genes, was conferred by the production of CTX-M-15 in association with frameshift mutations predicted in the OmpK35 and OmpK36 porin sequences, as previously described (16). The same mechanisms could also contribute to the slight increase of the CZA MICs observed in these strains, since no other known resistance mechanisms were detected in their genomes. The *bla*_CTX-M-15_ gene was located on pKPN in strain 1203, integrated into the chromosome. These strains belonged to the same subclade of strain 3 that developed MEM resistance by the acquisition of *bla*_KPC-3_ on pKpQIL. The gain of this plasmid requires a fully functional OmpK36 for conjugation (17). Yet, given the predicted depletion of this protein in isolates 1802 and 1203, the ancestor of pKpQIL-positive strains was presumably an isolate akin to 1802 or 1203, with a wild-type OmpK36 porin.

ST307 isolates gained CZA resistance by the acquisition of *bla*_KPC-31_ located on pKpQIL plasmids. In the historical ST307 strains of our collection, pKpQIL was observed in fusion with pKPN. In the recent isolates, *bla*_KPC-31_ was still located on pKpQIL, but the plasmid had evolved by transposition, recombination, and deletion events, associated with the loss of self-transmissibility.

Among the CZA-resistant isolates, strain 0323-2021 reached high resistance levels to both CZA and carbapenems by acquisition of three copies of Tn*4401a* on the same pKpQIL, carrying *bla*_KPC-31_ and *bla*_KPC-3_ genes. However, this configuration was unstable. In the same strain, smaller 83-kb pKpQIL versions were observed, probably due to homologous recombination between directly oriented Tn*4401a* copies, leading to recombined plasmids carrying Tn*4401a::bla*_KPC-31_ or Tn*4401a:bla*_KPC-3_ copies, respectively.

The 83-kb pKpQIL, carrying the Tn*4401a::bla*_KPC-31_ copy, was identified in strain 1001-2021 isolated from a blood culture from the same patient from whom strain 0323-2021 was recovered. This is an important finding because it demonstrates that the recombination and segregation of the 83-kb pKpQIL is not an artifact that arose in transformants *in vitro*; instead, it was an event that occurred in the patient *in vivo*. This plasmid confers CZA resistance, but the loss of the Tn*4401a::bla*_KPC-3_ copy is expected to restore susceptibility to MEM. However, strain 1001-2021 still showed an MEM MIC of 8 mg/L, probably due to the lack of the OmpK36 porin, that was not observed in the other KPC-31-producing strains, which showed lower MICs for carbapenems.

It should be noted that plasmids from both strains 1001-2021 and 0323-2021 all lacked a complete transfer locus because of the observed rearrangements. Consequently, the identified plasmids are expected not to be self-conjugative and cannot be transferred to other bacteria but can be propagated vertically within the ST307 clone.

The genetic complexity of the bacteria described in this study needed a lot of experimental work to be correctly described. It was not discerned by standard whole-genome sequencing (WGS) but was also at the limit of resolution of the hybrid ONT-Illumina combined approach.

Accurate molecular methods and monitoring of phenotypical changes can help us trace the elevated capacity of high-risk clones to evolve toward resistance under positive selection. Still, the careful use of novel antibiotics is a crucial action to prevent the development of resistance to life-saving antimicrobial agents in these highly flexible clones.

## MATERIALS AND METHODS

### Strain isolation and susceptibility testing.

Bacteria were isolated from samples processed for routine diagnostics; the species was identified by the matrix-assisted laser desorption ionization–time of flight mass spectrometry (MALDI-TOF MS) system (Bruker Daltonik GmbH, Bremen, Germany). Antimicrobial susceptibility was tested using the MicroScan WalkAway system (Beckman Coulter Inc., Brea, CA, USA), and the CZA and meropenem-vaborbactam (MVA) MICs were confirmed using Etest strips (Liofilchem, Roseto degli Abruzzi, Italy). The cefiderocol MICs were tested using the ComASP compact antimicrobial susceptibility panel for 0.008 to 128 mg/L cefiderocol (Liofilchem). All CZA-resistant strains were tested for *bla*_KPC_ genes (GeneXpert, Cepheid, CA, USA).

### ST307 analyzed in this study.

Strains 0213-2021, 0323-2021, and 1001-2021 were isolated from two patients hospitalized at the neurological surgery unit of PUI hospital (Table 1). A historical ST307 collection from 2019 was added to the study for comparison and included the following: strains 0603, 1203, and 1802, not producing carbapenemases; the KPC-3-producing strain 3; and the KPC-31-producing strains 27B and 21. Strains 27B and 21 were representative of the 5 CZA-resistant ST307 strains identified in 2019 at PUI (13). All strains resistant to CZA were susceptible to MVA (MIC, <0.2 mg/L) and cefiderocol (MIC, ≤2 mg/L).

### Whole-genome sequencing.

Whole-genome sequencing (WGS) was carried out on purified genomic DNA (Macherey-Nagel DNA extraction kit; Düren, Germany) using Illumina MiSeq (San Diego, CA, USA) sequencing. The Illumina reads were assembled through the public European Galaxy server (https://usegalaxy.eu/) using the SPAdes v3.15.3 pipeline (18). Oxford Nanopore Technologies (ONT) sequencing was performed as previously described (13). One round of polishing of the ONT reads was carried out using the Flye assembly (19), while short-read assembly was automatically performed using the SPAdes assembler in the Galaxy wrapper of the Unicycler v0.4.8.0 tool. Hybrid assembly was performed using normal bridging mode (20), and standard polishing parameters (lowest k-mer size, expressed as a fraction of the read length, of 0.2; filtering out contigs lower than 0.25 of the chromosomal depth) were adopted. The Staramr tool (21) was used on the resulting assemblies to detect antimicrobial resistance genes. The BacAnt tool (22) was used for annotation of transposable elements.

Plasmid DNA from 100 mL LB liquid broth cultures of E. coli DH5-α transformants and K. pneumoniae strains was purified using a plasmid midiprep purification kit (PureYield; Promega Italia Srl, Milan, Italy) and concentrated using Microcon 100 centrifugal filters (Merck KGaA, Darmstadt, Germany). Plasmid libraries were prepared with 400 ng of purified plasmid DNA and sequenced using ONT. The ONT reads were assembled using the Flye tool (19).

### Phylogenetic analysis.

A total of 2,040 completely assembled K. pneumoniae genomes were downloaded from the public NCBI data set (https://www.ncbi.nlm.nih.gov/datasets/docs/v1/how-tos/genomes/download-genome/). The Kleborate tool (23) was used to assess the STs of the downloaded sequences, and 30 genomes belonging to ST307 were retrieved and annotated using Prokka v1.14.6 (24). The resulting general feature formats (GFFs) were analyzed using Roary v3.11.3 (25). The core genome was defined as the genes found in all 39 isolates (25). Removal of recombining regions from the pangenome produced by Roary was carried out using Gubbins (26), generating a maximum likelihood phylogenetic tree using RAxML with default parameters (27). The tree and metadata were visualized with Microreact (28) and adjusted using the open-source software Inkscape.

### Plasmid transformation.

Purified plasmid DNA was obtained from overnight 50-mL LB liquid broth cultures of K. pneumoniae isolates. Plasmid extraction was performed using the PureYield plasmid midiprep system (Promega Italia Srl). Plasmid DNA was transformed into chemically competent Escherichia coli DH5-α cells (Life Technologies, Thermo Fisher Scientific, Waltham, MA, USA), selecting transformants on LB agar plates containing ceftazidime (CAZ, 6 mg/L). After 24 h, colonies were screened for *bla*_KPC_ using the KPC_FW_ and KPC_RV_ primer pair, as previously described (13).

The CAZ and MEM MICs of the KPC-positive E. coli DH5α transformants were tested using microdilution (MicroScan system; Beckman Coulter Inc.). The CZA MICs were determined using Etest strips (Liofilchem).

The plasmid content of transformants showing different CZA and MEM resistance was sequenced using ONT. To confirm the position of *bla*_KPC-31_ and *bla*_KPC-3_ at the Tn*4401* copies flanking the *traS* and *traN* genes, respectively, PCR was performed with the KPC_INT_/TraN_RV_ and KPC_INT_/TraS_FW_ primer pairs, followed by nested PCR performed with the KPC_INT_ and KPC_RV_ primer pair. The *bla*_KPC_ amplicons were sequenced using the Sanger method with both the KPC_INT_ and KPC_RV_ primers (see Table S1 in the supplemental material).

### Ethics.

Procedures performed in the study were in accordance with the ethical standards of the Institutional and National Research Committee and with the 1964 Helsinki Declaration and its later amendments or comparable ethical standards.

### Data availability.

The whole-genome sequences of the strains analyzed in this study are available under BioProject accession numbers PRJNA802088 (strains 0213-2021, 1001-2021, 0323-2021, 0603, and 1203) and PRJNA557206 (strain 1802). The complete sequences obtained in this study are available at NCBI GenBank under the following accession numbers: OM489434 (pKPN-1802), OM489428 (pKPN-1203; in the chromosome), ON002622.2 (pKpQIL-pKPN-21), OM489431 (pKpQIL-0213), OM489432 (pKPN-0213), OM489426 (pKpQIL-0323), OM489427 (pKPN-0323), OM489425 (pKpQIL-1001), OM489433 (pKPN-1001), ON002623.2 (pKpQIL-3), and ON002624 (pKPN-3). The genomes of strains 27B, 21, and 3 (BioProject accession number PRJNA648909) and plasmid pKpQIL-pKPN-27B (GenBank accession number MW650887) were previously described (13) and used in this study for comparison.

## References

[1] Boucher HW, Talbot GH, Bradley JS, Edwards JE, Gilbert D, Rice LB, Scheld M, Spellberg B, Bartlett J. 2009. Bad bugs, no drugs: no ESKAPE! An update from the Infectious Diseases Society of America. Clin Infect Dis 48:1–12. doi:10.1086/595011. 19035777

[2] Navon-Venezia S, Kondratyeva K, Carattoli A. 2017. *Klebsiella pneumoniae*: a major worldwide source and shuttle for antibiotic resistance. FEMS Microbiol Rev 41:252–275. doi:10.1093/femsre/fux013. 28521338

[3] Pitout JDD, Nordmann P, Poirel L. 2015. Carbapenemase-producing *Klebsiella pneumoniae*, a key pathogen set for global nosocomial dominance. Antimicrob Agents Chemother 59:5873–5884. doi:10.1128/AAC.01019-15. 26169401 PMC4576115

[4] Woodford N, Turton JF, Livermore DM. 2011. Multiresistant Gram-negative bacteria: the role of high-risk clones in the dissemination of antibiotic resistance. FEMS Microbiol Rev 35:736–755. doi:10.1111/j.1574-6976.2011.00268.x. 21303394

[5] Giani T, Pini B, Arena F, Conte V, Bracco S, Migliavacca R, Pantosti A, Pagani L, Luzzaro F, Rossolini GM, AMCLI-CRE Survey Participants. 2013. Epidemic diffusion of KPC carbapenemase-producing *Klebsiella pneumoniae* in Italy: results of the first countrywide survey, 15 May to 30 June 2011. Euro Surveill 18:20489. 23787077

[6] Leavitt A, Chmelnitsky I, Ofek I, Carmeli Y, Navon-Venezia S. 2010. Plasmid pKpQIL encoding KPC-3 and TEM-1 confers carbapenem resistance in an extremely drug-resistant epidemic *Klebsiella pneumoniae* strain. J Antimicrob Chemother 65:243–248. doi:10.1093/jac/dkp417. 19939824

[7] Peirano G, Chen L, Kreiswirth BN, Pitout JDD. 2020. Emerging antimicrobial-resistant high-risk *Klebsiella pneumoniae* clones ST307 and ST147. Antimicrob Agents Chemother 64:e01148-20. doi:10.1128/AAC.01148-20. 32747358 PMC7508593

[8] Wyres KL, Hawkey J, Hetland MAK, Fostervold A, Wick RR, Judd LM, Hamidian M, Howden BP, Löhr IH, Holt KE. 2019. Emergence and rapid global dissemination of CTX-M-15-associated *Klebsiella pneumoniae* strain ST307. J Antimicrob Chemother 74:577–581. doi:10.1093/jac/dky492. 30517666 PMC6376852

[9] Castanheira M, Farrell SE, Wanger A, Rolston KV, Jones RN, Mendes RE. 2013. Rapid expansion of KPC-2-producing *Klebsiella pneumoniae* isolates in two Texas hospitals due to clonal spread of ST258 and ST307 lineages. Microb Drug Resist 19:295–297. doi:10.1089/mdr.2012.0238. 23530541

[10] Villa L, Feudi C, Fortini D, Brisse S, Passet V, Bonura C, Endimiani A, Mammina C, Ocampo AM, Jimenez JN, Doumith M, Woodford N, Hopkins K, Carattoli A. 2017. Diversity, virulence, and antimicrobial resistance of the KPC-producing *Klebsiella pneumoniae* ST307 clone. Microb Genom 3:e000110. doi:10.1099/mgen.0.000110. 28785421 PMC5506382

[11] Lam MMC, Wick RR, Wyres KL, Gorrie CL, Judd LM, Jenney AWJ, Brisse S, Holt KE. 2018. Genetic diversity, mobilisation and spread of the yersiniabactin-encoding mobile element ICEKp in *Klebsiella pneumoniae* populations. Microb Genom 4:e000196. doi:10.1099/mgen.0.000196. 29985125 PMC6202445

[12] Shirley M. 2018. Ceftazidime-avibactam: a review in the treatment of serious Gram-negative bacterial infections. Drugs 78:675–692. doi:10.1007/s40265-018-0902-x. 29671219

[13] Carattoli A, Arcari G, Bibbolino G, Sacco F, Tomolillo D, Di Lella FM, Trancassini M, Faino L, Venditti M, Antonelli G, Raponi G. 2021. Evolutionary trajectories toward ceftazidime-avibactam resistance in *Klebsiella pneumoniae* clinical isolates. Antimicrob Agents Chemother 65:e0057421. doi:10.1128/AAC.00574-21. 34339281 PMC8448114

[14] Shields RK, Chen L, Cheng S, Chavda KD, Press EG, Snyder A, Pandey R, Doi Y, Kreiswirth BN, Nguyen MH, Clancy CJ. 2017. Emergence of ceftazidime-avibactam resistance due to plasmid-borne bla_KPC-3_ mutations during treatment of carbapenem-resistant *Klebsiella pneumoniae* infections. Antimicrob Agents Chemother 61:e02097-16. doi:10.1128/AAC.02097-16. 28031201 PMC5328542

[15] Campos-Madueno EI, Moser AI, Jost G, Maffioli C, Bodmer T, Perreten V, Endimiani A. 2022. Carbapenemase-producing *Klebsiella pneumoniae* strains in Switzerland: human and non-human settings may share high-risk clones. J Glob Antimicrob Resist 28:206–215. doi:10.1016/j.jgar.2022.01.016. 35085791

[16] García-Fernández A, Miriagou V, Papagiannitsis CC, Giordano A, Venditti M, Mancini C, Carattoli A. 2010. An ertapenem-resistant extended-spectrum-beta-lactamase-producing *Klebsiella pneumoniae* clone carries a novel OmpK36 porin variant. Antimicrob Agents Chemother 54:4178–4184. doi:10.1128/AAC.01301-09. 20660683 PMC2944588

[17] Low WW, Wong JLC, Beltran LC, Seddon C, David S, Kwong H-S, Bizeau T, Wang F, Peña A, Costa TRD, Pham B, Chen M, Egelman EH, Beis K, Frankel G. 2022. Mating pair stabilization mediates bacterial conjugation species specificity. Nat Microbiol 7:1016–1027. doi:10.1038/s41564-022-01146-4. 35697796 PMC9246713

[18] Bankevich A, Nurk S, Antipov D, Gurevich AA, Dvorkin M, Kulikov AS, Lesin VM, Nikolenko SI, Pham S, Prjibelski AD, Pyshkin AV, Sirotkin AV, Vyahhi N, Tesler G, Alekseyev MA, Pevzner PA. 2012. SPAdes: a new genome assembly algorithm and its applications to single-cell sequencing. J Comput Biol 19:455–477. doi:10.1089/cmb.2012.0021. 22506599 PMC3342519

[19] Freire B, Ladra S, Parama JR. 2021. Memory-efficient assembly using Flye. IEEE/ACM Trans Comput Biol Bioinform 19:3564–3577. doi:10.1109/TCBB.2021.3108843. 34469305

[20] Wick RR, Judd LM, Gorrie CL, Holt KE. 2017. Unicycler: resolving bacterial genome assemblies from short and long sequencing reads. PLoS Comput Biol 13:e1005595. doi:10.1371/journal.pcbi.1005595. 28594827 PMC5481147

[21] Bharat A, Petkau A, Avery BP, Chen JC, Folster JP, Carson CA, Kearney A, Nadon C, Mabon P, Thiessen J, Alexander DC, Allen V, El Bailey S, Bekal S, German GJ, Haldane D, Hoang L, Chui L, Minion J, Zahariadis G, Domselaar GV, Reid-Smith RJ, Mulvey MR. 2022. Correlation between phenotypic and in silico detection of antimicrobial resistance in Salmonella enterica in Canada using Staramr. Microorganisms 10:292. doi:10.3390/microorganisms10020292. 35208747 PMC8875511

[22] Hua X, Liang Q, Deng M, He J, Wang M, Hong W, Wu J, Lu B, Leptihn S, Yu Y, Chen H. 2021. BacAnt: a combination annotation server for bacterial DNA sequences to identify antibiotic resistance genes, integrons, and transposable elements. Front Microbiol 12:649969. doi:10.3389/fmicb.2021.649969. 34367079 PMC8343408

[23] Lam MMC, Wick RR, Watts SC, Cerdeira LT, Wyres KL, Holt KE. 2021. A genomic surveillance framework and genotyping tool for *Klebsiella pneumoniae* and its related species complex. Nat Commun 12:4188. doi:10.1038/s41467-021-24448-3. 34234121 PMC8263825

[24] Seemann T. 2014. Prokka: rapid prokaryotic genome annotation. Bioinformatics 30:2068–2069. doi:10.1093/bioinformatics/btu153. 24642063

[25] Page AJ, Cummins CA, Hunt M, Wong VK, Reuter S, Holden MTG, Fookes M, Falush D, Keane JA, Parkhill J. 2015. Roary: rapid large-scale prokaryote pan genome analysis. Bioinformatics 31:3691–3693. doi:10.1093/bioinformatics/btv421. 26198102 PMC4817141

[26] Croucher NJ, Page AJ, Connor TR, Delaney AJ, Keane JA, Bentley SD, Parkhill J, Harris SR. 2015. Rapid phylogenetic analysis of large samples of recombinant bacterial whole genome sequences using Gubbins. Nucleic Acids Res 43:e15. doi:10.1093/nar/gku1196. 25414349 PMC4330336

[27] Stamatakis A. 2014. RAxML version 8: a tool for phylogenetic analysis and post-analysis of large phylogenies. Bioinformatics 30:1312–1313. doi:10.1093/bioinformatics/btu033. 24451623 PMC3998144

[28] Argimón S, Abudahab K, Goater RJE, Fedosejev A, Bhai J, Glasner C, Feil EJ, Holden MTG, Yeats CA, Grundmann H, Spratt BG, Aanensen DM. 2016. Microreact: visualizing and sharing data for genomic epidemiology and phylogeography. Microb Genom 2:e000093. doi:10.1099/mgen.0.000093. 28348833 PMC5320705

