# Supplementary material for: Multiplicity of blaKPC Genes and pKpQIL Plasmid Plasticity in the Development of Ceftazidime-Avibactam and Meropenem Coresistance in Klebsiella pneumoniae Sequence Type 307
Source: Antimicrob Agents Chemother. 2023 Jul 10;67(8):e00368-23. doi: 10.1128/aac.00368-23 (PMC10433805; doi:10.1128/aac.00368-23)
Supplement: Supplemental file 1 — Supplemental material. Download aac.00368-23-s0001.pdf, PDF file, 0.6 MB [file aac.00368-23-s0001.pdf]

Suppl. Table 1. Primers used in *Escherichia coli* DH5-α transformants analyzed in this study

Primer	Sequence	Expected product base pairs in sequence OM489426
KPC_{INT}	5’-CTGTCTTGTCTCTCATGGCC-3’	3142 bp<br>Position nt 74148-77290
$TraN_{RV}$	5’-GGGGATGCCAGCCAGATAAA-3'	
KPC_{INT}	5’-CTGTCTTGTCTCTCATGGCC-3’	2910 bp<br>Position nt 87578-90488
$TraS_{FW}$	5’-CGAGACCTTCAAGACAAGTAA-3’	
KPC_{INT}	5’-CTGTCTTGTCTCTCATGGCC-3’	922 bp<br>Position nt 74148-75070<br>Position nt 89566-90488
KPC_{RV}	5’-ACAGTGGTTGGTAATCCATGC-3’	

snp-dists (	0213.gff3	0323.gff3	0603.gff3	1001.gff3	1203.gff3	1802.gff3	21.gff3	27B.gff3	3.gff3	GCA_0028	GCA_0028	GCA_0030	GCA_0035	GCA_0035	GCA_0035	GCA_0035	GCA_0039	GCA_0097	GCA_0129
0213.gff3	0	48	83	47	65	65	15	16	63	93	94	69	104	76	76	82	88	314	71
0323.gff3	48	0	95	1	77	77	45	46	75	105	106	81	116	88	88	94	100	326	83
0603.gff3	83	95	0	94	66	66	82	83	64	88	89	50	97	69	69	75	69	295	72
1001.gff3	47	1	94	0	76	76	44	45	74	104	105	80	115	87	87	93	99	325	82
1203.gff3	65	77	66	76	0	0	64	65	6	76	77	52	85	57	57	63	71	297	46
1802.gff3	65	77	66	76	0	0	64	65	6	76	77	52	85	57	57	63	71	297	46
21.gff3	15	45	82	44	64	64	0	7	62	92	91	68	103	75	75	81	87	313	70
27B.gff3	16	46	83	45	65	65	7	0	63	93	94	69	104	76	76	82	88	314	71
3.gff3	63	75	64	74	6	6	62	63	0	74	75	50	85	57	57	63	69	295	44
GCA_0028	93	105	88	104	76	76	92	93	74	0	15	74	109	81	81	87	90	316	82
GCA_0028	94	106	89	105	77	77	91	94	75	15	0	75	110	82	82	88	91	317	83
GCA_0030	69	81	50	80	52	52	68	69	50	74	75	0	85	57	57	63	47	271	58
GCA_0035	104	116	97	115	85	85	103	104	85	109	110	85	0	28	28	35	104	330	93
GCA_0035	76	88	69	87	57	57	75	76	57	81	82	57	28	0	0	7	76	302	65
GCA_0035	76	88	69	87	57	57	75	76	57	81	82	57	28	0	0	7	76	302	65
GCA_0035	82	94	75	93	63	63	81	82	63	87	88	63	35	7	7	0	82	308	71
GCA_0039	88	100	69	99	71	71	87	88	69	90	91	47	104	76	76	82	0	285	77
GCA_0097	314	326	295	325	297	297	313	314	295	316	317	271	330	302	302	308	285	0	303
GCA_0129	71	83	72	82	46	46	70	71	44	82	83	58	93	65	65	71	77	303	0
GCA_0129	84	96	79	95	67	67	83	84	65	89	90	65	82	54	54	60	84	310	73
GCA_0129	79	91	74	90	62	62	78	79	60	84	85	60	77	49	49	55	79	305	68
GCA_0129	71	83	66	82	54	54	70	71	52	75	76	52	83	55	55	61	71	297	59
GCA_0129	152	164	147	163	135	135	151	152	133	156	157	133	150	122	122	128	152	376	140
GCA_0129	79	91	74	90	62	62	78	79	60	84	85	60	47	19	19	26	79	305	68
GCA_0129	102	114	83	113	85	85	101	102	83	104	105	61	118	90	90	96	66	299	91
GCA_0129	77	89	72	88	60	60	76	77	58	82	83	58	75	47	47	53	77	302	66
GCA_0129	65	77	60	76	48	48	64	65	46	69	70	46	63	35	35	41	65	291	53
GCA_0129	94	106	89	105	77	77	93	94	75	99	100	75	92	64	64	69	94	320	83
GCA_0129	75	87	70	86	58	58	74	75	56	75	76	56	73	45	45	51	69	295	62
GCA_0129	102	114	83	113	85	85	101	102	83	104	105	61	118	90	90	96	66	299	91
GCA_0129	90	102	85	101	73	73	89	90	71	95	96	71	88	60	60	66	90	316	79
GCA_0129	69	81	64	80	52	52	68	69	50	74	75	50	67	39	39	45	69	294	58
GCA_0129	72	84	67	83	55	55	70	72	53	77	78	53	70	42	42	48	72	296	61
GCA_0133	74	86	75	85	41	41	73	74	39	85	86	61	96	68	68	74	80	306	55
GCA_0133	62	74	63	73	29	29	61	62	27	73	74	49	84	56	56	62	68	294	43
GCA_0134	97	108	46	107	80	80	96	97	78	102	103	64	110	82	82	88	83	309	86
GCA_0169	68	80	63	79	51	51	67	68	49	73	74	49	84	56	56	62	68	294	57
GCA_0176	114	126	95	125	97	97	113	114	95	119	119	73	130	102	102	108	84	317	103
GCA_0197	95	107	75	106	78	78	94	95	76	100	101	54	111	83	83	89	65	297	84

snp-dists (	GCA_0129	GCA_0129	GCA_0129	GCA_0129	GCA_0129	GCA_0129	GCA_0129	GCA_0129	GCA_0129	GCA_0129	GCA_0129	GCA_0129	GCA_0129	GCA_0129	GCA_0133	GCA_0133	GCA_0134	GCA_0169	GCA_0176	GCA_0197
0213.gff3	84	79	71	152	79	102	77	65	94	75	102	90	69	72	74	62	97	68	114	95
0323.gff3	96	91	83	164	91	114	89	77	106	87	114	102	81	84	86	74	108	80	126	107
0603.gff3	79	74	66	147	74	83	72	60	89	70	83	85	64	67	75	63	46	63	95	75
1001.gff3	95	90	82	163	90	113	88	76	105	86	113	101	80	83	85	73	107	79	125	106
1203.gff3	67	62	54	135	62	85	60	48	77	58	85	73	52	55	41	29	80	51	97	78
1802.gff3	67	62	54	135	62	85	60	48	77	58	85	73	52	55	41	29	80	51	97	78
21.gff3	83	78	70	151	78	101	76	64	93	74	101	89	68	70	73	61	96	67	113	94
27B.gff3	84	79	71	152	79	102	77	65	94	75	102	90	69	72	74	62	97	68	114	95
3.gff3	65	60	52	133	60	83	58	46	75	56	83	71	50	53	39	27	78	49	95	76
GCA_0028	89	84	75	156	84	104	82	69	99	75	104	95	74	77	85	73	102	73	119	100
GCA_0028	90	85	76	157	85	105	83	70	100	76	105	96	75	78	86	74	103	74	119	101
GCA_0030	65	60	52	133	60	61	58	46	75	56	61	71	50	53	61	49	64	49	73	54
GCA_0035	82	77	83	150	47	118	75	63	92	73	118	88	67	70	96	84	110	84	130	111
GCA_0035	54	49	55	122	19	90	47	35	64	45	90	60	39	42	68	56	82	56	102	83
GCA_0035	54	49	55	122	19	90	47	35	64	45	90	60	39	42	68	56	82	56	102	83
GCA_0035	60	55	61	128	26	96	53	41	69	51	96	66	45	48	74	62	88	62	108	89
GCA_0039	84	79	71	152	79	66	77	65	94	69	66	90	69	72	80	68	83	68	84	65
GCA_0097	310	305	297	376	305	299	302	291	320	295	299	316	294	296	306	294	309	294	317	297
GCA_0129	73	68	59	140	68	91	66	53	83	62	91	79	58	61	55	43	86	57	103	84
GCA_0129	0	57	63	130	57	98	55	43	72	53	96	40	47	49	76	64	93	64	110	91
GCA_0129	57	0	58	124	52	93	50	38	67	48	93	63	42	45	71	59	88	59	105	86
GCA_0129	63	58	0	131	58	85	56	44	73	54	85	68	48	51	63	51	80	51	97	78
GCA_0129	130	124	131	0	125	166	123	103	140	113	166	136	115	118	144	132	161	132	178	159
GCA_0129	57	52	58	125	0	93	50	38	67	48	93	63	42	45	71	59	87	59	105	86
GCA_0129	98	93	85	166	93	0	91	79	108	83	26	104	83	86	94	82	97	82	98	79
GCA_0129	55	50	56	123	50	91	0	36	65	46	91	61	32	35	69	57	86	57	103	84
GCA_0129	43	38	44	103	38	79	36	0	53	26	79	49	28	31	57	45	74	45	91	72
GCA_0129	72	67	73	140	67	108	65	53	0	63	108	78	57	60	86	74	103	74	120	101
GCA_0129	53	48	54	113	48	83	46	26	63	0	83	58	38	41	67	55	84	55	101	82
GCA_0129	96	93	85	166	93	26	91	79	108	83	0	104	83	85	94	82	97	82	98	79
GCA_0129	40	63	68	136	63	104	61	49	78	58	104	0	53	56	82	70	99	70	116	97
GCA_0129	47	42	48	115	42	83	32	28	57	38	83	53	0	23	61	49	78	49	95	76
GCA_0129	49	45	51	118	45	86	35	31	60	41	85	56	23	0	64	52	81	52	98	79
GCA_0133	76	71	63	144	71	94	69	57	86	67	94	82	61	64	0	14	89	60	106	87
GCA_0133	64	59	51	132	59	82	57	45	74	55	82	70	49	52	14	0	77	48	94	75
GCA_0134	93	88	80	161	87	97	86	74	103	84	97	99	78	81	89	77	0	76	109	89
GCA_0169	64	59	51	132	59	82	57	45	74	55	82	70	49	52	60	48	76	0	94	75
GCA_0176	110	105	97	178	105	98	103	91	120	101	98	116	95	98	106	94	109	94	0	91
GCA_0197	91	86	78	159	86	79	84	72	101	82	79	97	76	79	87	75	89	75	91	0

Supplementary Dataset 1. Distance matrix among the genomes analyzed in this study. Numbers represent Single Nucleotide Polymorphisms (SNPs) identified by SNP Distance Matrix algorithm.

	bla KPC-3	*bla* KPC-31	*bla* CTX-M-15	*bla* OXA-1	*bla* TEM-1B	*bla* TEM-1A	*aac(3)-IIe*	*aac(6')-Ib-cr*	*aph(3'')-Ib*	*aph(6)-Id*	*ΔcatB3*	*dfrA14*	*qnrB1*	*sul2*	*tet* (A)
1802	neg	neg	pos	pos	pos	neg	pos	pos	pos	pos	pos	pos	pos	pos	pos
1203	neg	neg	pos	pos	pos	neg	pos	pos	pos	pos	pos	pos	pos	pos	pos
21	neg	pos	pos	pos	pos	neg	pos	pos	pos	pos	pos	pos	pos	pos	pos
27B	neg	pos	pos	pos	pos	neg	pos	pos	pos	pos	pos	pos	pos	pos	pos
0213	neg	pos	pos	pos	neg	pos	pos	pos	pos	pos	pos	pos	pos	pos	pos
0323	pos	pos	pos	pos	neg	pos	pos	pos	pos	pos	pos	pos	pos	pos	pos
1001	neg	pos	pos	pos	neg	pos	pos	pos	pos	pos	pos	pos	pos	pos	pos
3	pos	neg	pos	pos	neg	pos	pos	pos	pos	pos	pos	pos	pos	pos	neg
0603	neg	neg	pos	pos	neg	neg	pos	pos	neg	neg	pos	pos	pos	neg	neg

Supplementary Dataset 2. Presence (pos) or absence (neg) of resistance genes identified by ResFinder in the ST307 *Klebsiella pneumoniae* genomes analyzed in this study

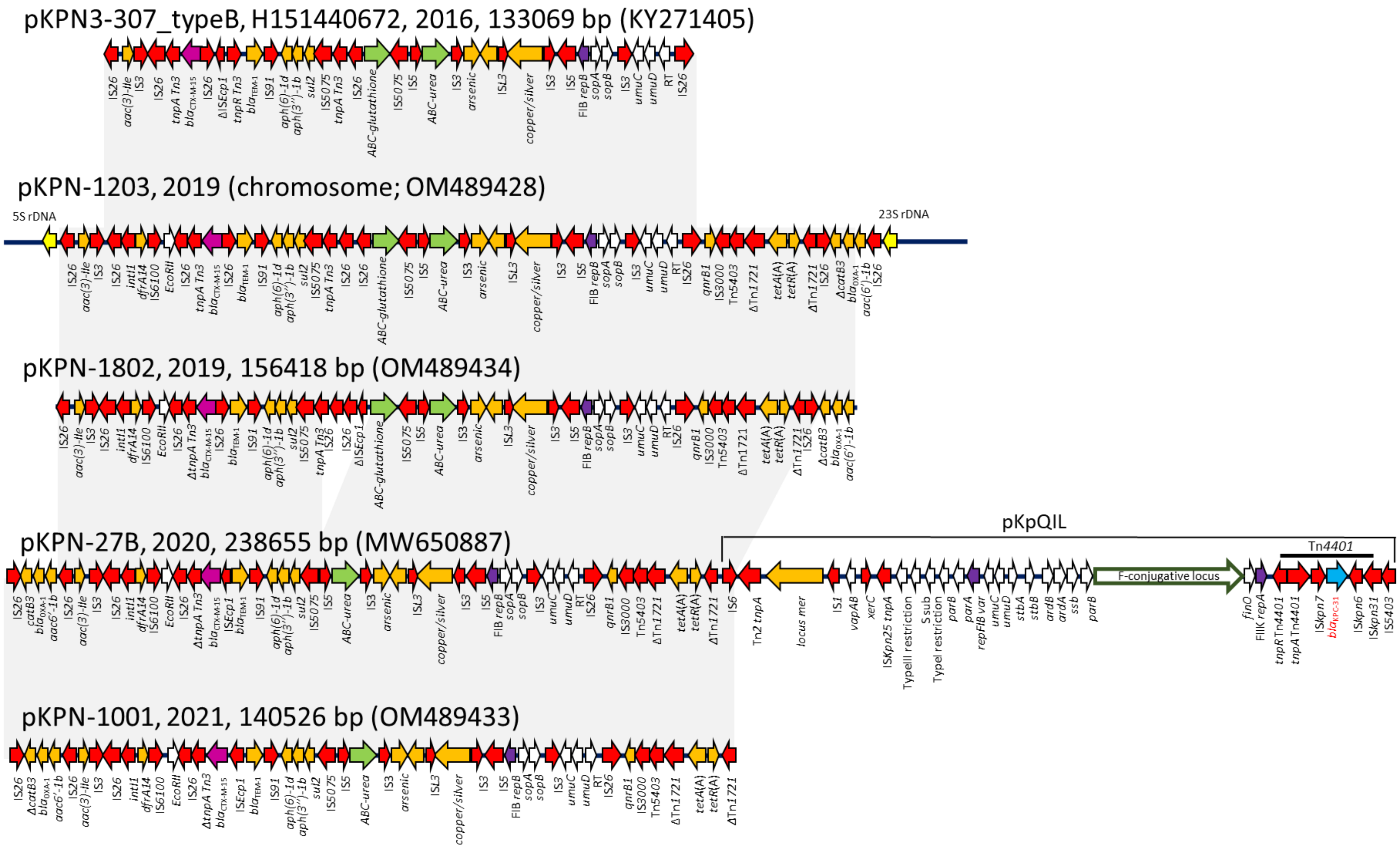

Supplementary Figure 1. **Plasmid maps of pKPN-307 plasmids.** Major structural features of pKPN plasmids identified in ST307 *K. pneumoniae* isolates H151440672 (reference), 1203, 1802, 27B and 1001-2021, respectively. Predicted coding sequences are indicated by colored arrows oriented in the direction of transcription of each respective gene: resistance genes, yellow; transposon-related genes and insertion sequences, red; replicons, violet; ABC uptake clusters, green; chromosomal 5S/23S rDNA genes, light yellow; other genes, white. The sizes of the arrows are not to scale.

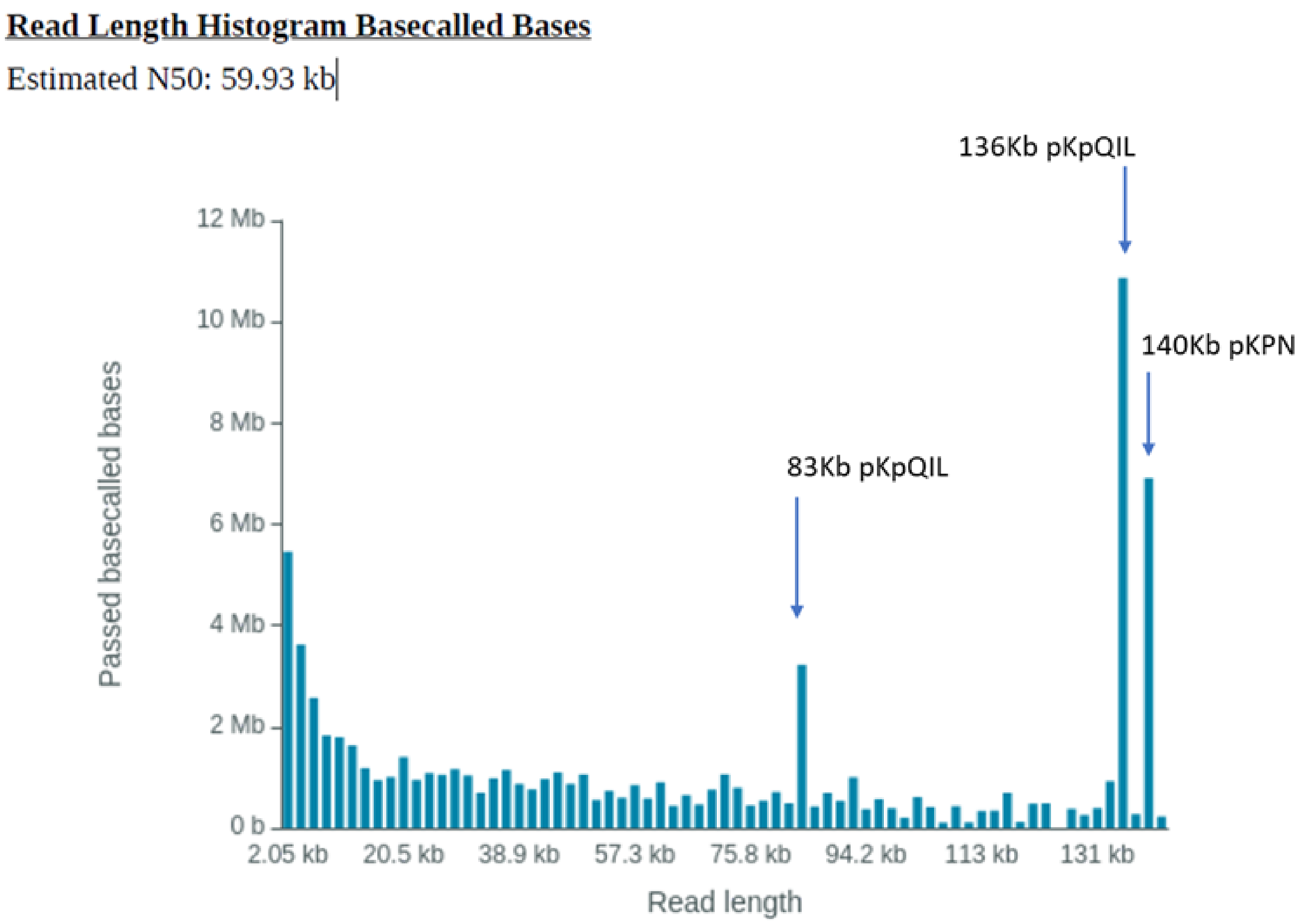

Supplementary Figure 2. Oxford Nanopore Technologies sequencing of purified plasmid DNAs from ST307 *Klebsiella pneumoniae* strain 0323. Read Length Histogram Basecalled Bases obtained by sequencing the purified plasmid DNA from the 0323-2021 *K. pneumoniae* strain into the MinION Flow Cell (R9.4.1) following ONT procedures. The read-length graph shows the total number of bases *vs* the read length. Arrows indicate the bars corresponding to single reads covering the entire length of the 83-Kb and 136-Kb pKpQIL plasmid derivatives and the 140-Kb pKPN plasmid co-resident with the 0323-2021 *K. pneumoniae* strain.

Strain	KPC	MEM[1]	CZA[1]	CAZ[1]
**3-1-TR**	KPC-3	1	<2	8
**0323-1-TR**	KPC-3	1	<2	8
**0323-11-TR**	KPC-31	<0.12	4	16
**0323-37-TR**	KPC-3/31	4	4	>32
**DH5-α**	Neg	<0.12	<2	≤1

Suppl. Table 2. Minimal inhibitory concentrations for carbapenem, and ceftazidime-avibactam in *Escherichia coli* DH5-α transformants analyzed in this study

[1]Minimal Inhibitory concentrations for MEM: Meropenem, CAZ: Ceftazidime; CZA: Ceftazidime-Avibactam. Neg: No KPC present.

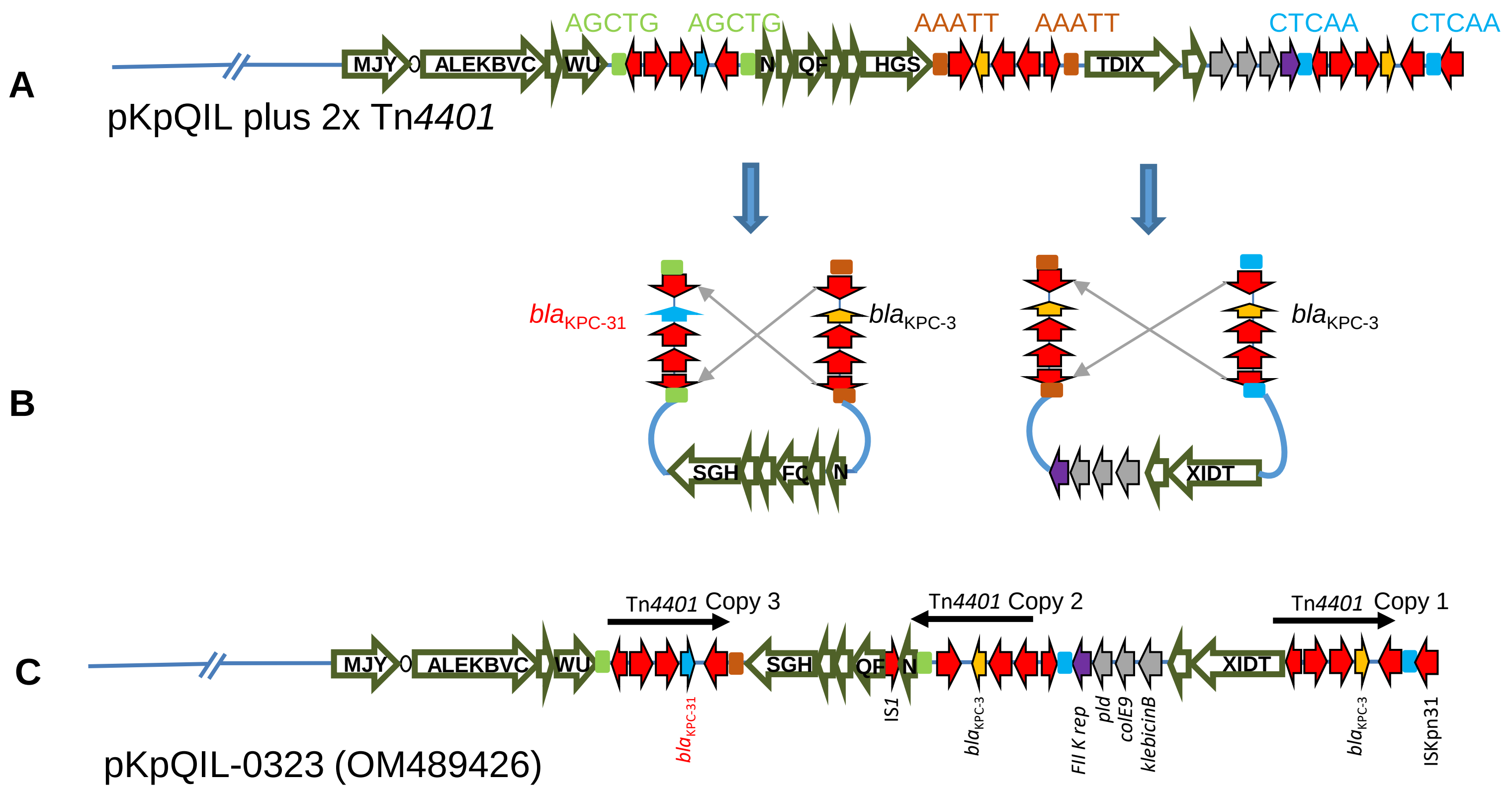

Supplementary Figure 3. Tn*4401* rearrangements in pKpQIL

Panel A: a canonical pKpQIL plasmid (only the regions of interest are represented) is drawn to design the three integration sites and the relative 5-bp target-site duplication, indicated with dark blu, brown and green boxes, due to the transposition of the three copies of the Tn*4401*::*bla*$_{KPC}$.

Panel B: the expected recombination occurred between Tn*4401*::*bla*$_{KPC}$ copies in opposite orientation (copies 3 and 2 and copies 1 and 3)

Panel C: inversions caused by recombination occurred between Tn*4401*::*bla*$_{KPC}$ copies resulting in the pKpQIL-0323 map. The rearrangements have been hypothesized by analysing the position of the 5-bp target-site duplication in the sequenced pKpQIL-0323 plasmid, also considering that , inversions changed them in reverse-complement sequences and moved far from the original integration site. The sizes of the arrows are not to scale.
